# Disease association with two *Helicobacter pylori *duplicate outer membrane protein genes, *homB *and *homA*

**DOI:** 10.1186/1757-4749-1-12

**Published:** 2009-06-22

**Authors:** Monica Oleastro, Rita Cordeiro, Yoshio Yamaoka, Dulciene Queiroz, Francis Mégraud, Lurdes Monteiro, Armelle Ménard

**Affiliations:** 1 Departamento de Doenças Infecciosas, Instituto Nacional Saúde Dr Ricardo Jorge, Av. Padre Cruz, 1649-016 Lisboa, Portugal; 2 INSERM U853, 33076 Bordeaux, France; 3 Department of Medicine, Michael E. DeBakey Veterans Affairs Medical Center and Baylor College of Medicine, 2002 Holcombe Blvd., Houston, Texas 77030, USA; 4 Laboratório de Pesquisa Bacteriologia, Faculdade de Medicina, UFMG, Av. Alfredo Balena, 190 S/4026 30130-100, Belo Horizonte, Brazil; 5 Université *Victor Segalen *Bordeaux 2, Laboratoire de Bactériologie, Bat. 2B RDC Zone Nord, 33076 Bordeaux cedex, France

## Abstract

**Background:**

*homB *encodes a *Helicobacter pylori *outer membrane protein. This gene was previously associated with peptic ulcer disease (PUD) and was shown to induce activation of interleukin-8 secretion *in vitro*, as well as contributing to bacterial adherence. Its 90%-similar gene, *homA*, was previously correlated with gastritis. The present study aimed to evaluate the gastric disease association with *homB *and *homA*, as well as with the *H. pylori *virulence factors *cagA*, *babA *and *vacA*, in 415 *H. pylori *strains isolated from patients from East Asian and Western countries. The correlation among these genotypes was also evaluated.

**Results:**

Both *homB *and *homA *genes were heterogeneously distributed worldwide, with a marked difference between East Asian and Western strains. In Western strains (n = 234, 124 PUD and 110 non-ulcer dyspepsia (NUD), *homB*, *cagA *and *vacA *s1 were all significantly associated with PUD (p = 0.025, p = 0.014, p = 0.039, respectively), and *homA *was closely correlated with NUD (p = 0.072). In East Asian strains (n = 138, 73 PUD and 65 NUD), *homB *was found more frequently than *homA*, and none of these genes was associated with the clinical outcome.

Overall, *homB *was associated with the presence of *cagA *(p = 0.043) and *vacA *s1 (p < 0.001), whereas *homA *was found more frequently in *cagA*-negative (p = 0.062) and *vacA *s2 (p < 0.001) strains.

Polymorphisms in *homB *and *homA *copy number were observed, with a clear geographical specificity, suggesting an involvement of these genes in host adaptation. A correlation between the *homB *two-copy genotype and PUD was also observed, emphasizing the role of *homB *in the virulence of the strain.

**Conclusion:**

The global results suggest that *homB *and *homA *contribute to the determination of clinical outcome.

## Background

*Helicobacter pylori *colonization of the human stomach is associated with chronic gastritis and an increased risk of peptic ulcer disease (PUD), gastric adenocarcinoma and gastric mucosa-associated lymphoid tissue lymphoma [1-3]. While some *H. pylori*-infected individuals remain asymptomatic, others develop severe gastric disease. Strain-dependent factors may account for differences in clinical outcome, in particular factors that modulate interactions between *H. pylori *and human gastric cells, such as outer membrane proteins (OMP) [4-6]. Recently, the OMP coding gene *homB *was associated with an increased risk of PUD in Portuguese children and young adults (age < 40 years) [7]. Moreover, *in vitro *assays showed that HomB contributes to the proinflammatory characteristics of *H. pylori *and is involved in bacterial adherence, these two phenomena being more pronounced when *homB *is present in two copies in a given strain, compared to one copy only [7]. The *homB *90%-similar gene, designated *homA*, was found to be associated with non-ulcer dyspepsia (NUD) in that same population [7].

In this study, we investigated gastric disease association with *homB *and *homA*, as well as the *H. pylori *virulence factors *cagA*, *vacA *and *babA*, in a panel of *H. pylori *clinical strains isolated from patients from East Asian and Western countries, presenting different gastric diseases, namely NUD and PUD. The correlation between those bacterial factors was also evaluated.

## Results

The presence of *homB *and *homA *in the *H. pylori *clinical strains was determined by PCR. Table 1 summarizes the characteristics of the study population. PCR products, corresponding to either *homA*, *homB *or both genes were obtained for all the 415 strains tested. The presence of both genes in the same genome was detected in 43 strains (10.4%) (36 PUD strains and 7 NUD strains). These strains were excluded from the analysis related to clinical outcome. Thus, a total of 372 strains were included. They comprised 197 strains isolated from PUD patients (66.3% male; 50.3 ± 14.5 years) and 175 strains isolated from NUD patients (53.7% male; 51.1 ± 13.4 years).

**Table 1:** Distribution of *Helicobacter pylori *strains included in the study (n = 415), according to geographical origin and disease status of patients.

Origin	Disease	No. of strains	Gender (% male)	Median age ± SD (years)
**Western countries**				

Portugal	NUD	50	44.7	51.3 ± 14.6
	DU	36	44.4	47.6 ± 16.6
	GU	14	76.9	54.8 ± 14.1
	Total number	100	47.3	51.2 ± 15.1
France	NUD	6	100.0	38.0 ± 7.8
	DU	28	80.0	49.3 ± 14.3
	Total number	34	82.9	47.7 ± 14.1
Sweden	NUD	10	28.6	62.1 ± 6.6
	DU	17	80.0	69.7 ± 12.9
	Total number	27	58.8	66.6 ± 11.2
Germany	NUD	10	40.0	57.3 ± 11.0
	DU	10	60.0	59.8 ± 13.3
	Total number	20	50.0	58.6 ± 11.9
USA	NUD	14	57.1	41.3 ± 8.8
	DU	15	73.3	55.6 ± 10.5
	Total number	29	67.9	48.7 ± 12.0
Brazil	NUD	18	45.0	49.3 ± 13.4
	DU	19	35.0	50.0 ± 18.8
	Total number	37	52.4	49.7 ± 15.7
Colombia	NUD	9	30.0	53.0 ± 13.6
	DU	10	88.9	46.7 ± 11.5
	Total number	19	57.9	50.0 ± 12.7

**East Asian countries**				

Japan	NUD	28	46.7	55.8 ± 16.1
	DU	22	59.1	40.6 ± 11.5
	GU	21	76.2	54.4 ± 12.1
	Total number	71	57.9	44.3 ± 12.7
South Korea	NUD	37	79.5	46.4 ± 10.6
	DU	29	70.8	45.8 ± 11.9
	GU	1	*	-
	Total number	67	76.1	44.7 ± 9.9

**African country**				

Burkina Faso	DU	11	N.A.	N.A.

NUD, non-ulcer dyspepsia

DU, duodenal ulcer

GU, gastric ulcer

No., number

N.A. data not available

* 38 year old male patient

### Distribution of *homB *and *homA *according to clinical outcome

**Figure 1:**
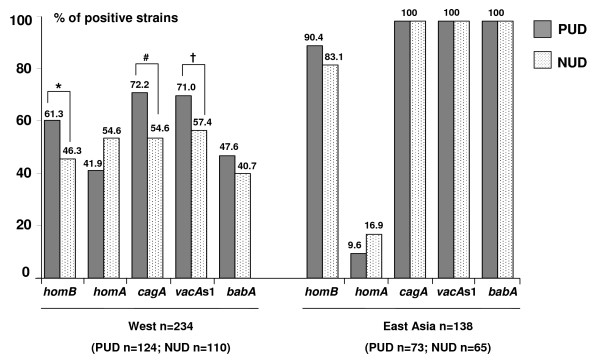
**Distribution of *Helicobacter pylori *genotypes among 372 strains isolated from East Asian and Western countries with regard to clinical outcome**. PUD (peptic ulcer disease). NUD (non-ulcer dyspepsia). *p = 0.025; OR = 1.84, 95%CI [1.10–3.10]. ^#^p = 0.014; OR = 2.2, 95%CI [1.20–3.86]. ^†^p = 0.039; OR = 1.8, 95%CI [1.05–3.12].

The results comparing PUD and NUD strains (n = 372) in different countries are presented in Fig. 1. Overall, *homB *was significantly more prevalent in PUD than in NUD strains (75.9 vs 64.7%, p = 0.026, OR = 1.7, 95%CI [1.06–2.74]), a trend also observed in Western strains (61.3 vs 46.3%, p = 0.025, OR = 1.84, 95%CI [1.10–3.10]). East Asian strains were predominantly *homB*-positive regardless of the clinical outcome (90.4% in PUD and 83.1% in NUD). Considering the analysis by country (Table 2), *homB *was associated with PUD in strains from France, Sweden, Brazil and Colombia, although with no statistical significance.

**Table 2:** Univariate analysis of the relationship between *Helicobacter pylori *virulence genotypes and clinical outcome according to country, from patients presenting peptic ulcer disease or non-ulcer dyspepsia.

	Prevalence (%) PUD vs NUD p-value^†^; OR [95%CI]				
	
	*homB*	*homA*	*cagA*	*vac*A s1	*babA*
Portugal 50 PUD; 50 NUD	59.6 vs 56.0 N.S.	40.4 vs 44.0 N.S.	**72.3 vs 40.0** **0.002; 1.88 [1.26–2.81]**	63.8 vs 44.0 N.S.	48.9 vs 32.0 N.S.
France 27 PUD; 6 NUD	74.1 vs 28.6 N.S.	37.0 vs 71.4 N.S.	85.2 vs 60.0 N.S.	85.2 vs 80.0 N.S.	80.0 vs 50.0 N.S.
Sweden 12 PUD; 10 NUD	41.7 vs 30.0 N.S.	58.3 vs 70 N.S.	83.3 vs 70.0 N.S.	66.7 vs 70.0 N.S.	83.3 vs 40.0 N.S.
Germany 9 PUD; 10 NUD	55.6 vs 60.0 N.S.	44.4 vs 40.0 N.S.	100 vs 80 N.S.	100 vs 80.0 N.S.	77.8 vs 60.0 N.S.
USA 10 PUD; 13 NUD	40.0 vs 33.3 N.S.	60.0 vs 66.7 N.S.	60.0 vs 91.7 N.S.	70.0 vs 91.7 N.S.	100 vs 91.7 N.S.
Brazil 10 PUD; 12 NUD	70.0 vs 27.3 N.S.	30.0 vs 72.7 N.S.	60 vs 18.2 N.S.	50.0 vs 27.3 N.S.	100 vs 100 N.S.
Colombia 9 PUD; 9 NUD	77.8 vs 60.0 N.S.	33.3 vs 50.0 N.S.	55.6 vs 80 N.S.	66.7 vs 70.0 N.S.	100 vs 70.0 N.S.
Japan 42 PUD; 28 NUD	95.5 vs 92.9 N.S.	4.7 vs 3.6 N.S.	100 vs 100 N.A	100 vs 100 N.A	100 vs 100 N.A
South Korea 28 PUD; 37 NUD	83.3 vs 73.7 N.S.	16.7 vs 26.3 N.S.	100 vs 100 N.A	100 vs 100 N.A	100 vs 100 N.A

Among the 415 isolates initially included, 43 (36 PUD strains and 7 NUD) harbored both *homA *and *homB *genes and were excluded from further analyses. These 49 isolates comprised all the 11 isolates from Burkina Faso.

† p-value was determined by the Fisher's Exact Test.

OR, odds ratio.

N.S., not significant.

N.A, not applicable.

PUD, peptic ulcer disease

NUD, non-ulcer dyspepsia

Inversely, the *hom*A gene was more prevalent in gastritis than in ulcer strains (35.8 vs 25.8%, p = 0.046, OR = 1.61, 95%CI [1.06–2.74]), a trend also observed in Western strains (54.6 vs 41.9%), though not significant (Fig. 1). The analysis by country revealed that *homA *was more frequently detected in strains isolated from NUD than from PUD in strains from France, Sweden, Brazil and Colombia, although the difference was not statistically significant (Table 2). Previously, it had been shown that *homB *was strongly associated with PUD strains isolated from young adults (age < 40 years) [7]. In the present study, a total of 90 strains were isolated from this age group. In this group, *homB *was significantly associated with PUD (n = 47, mean age 35.7 ± 5.8 y, 47.8% men) when compared to NUD strains (n = 43, mean age 33.4 ± 5.2 y, 55.9% men) (78.7 vs 48.8%; p = 0.006, OR = 3.88, 95% CI [1.41–10.84]). When considering only Western strains (31 PUD and 28 NUD), the same association was found (74.2 vs 35.7%; p = 0.007, OR = 5.18, 95% CI [1.49–18.68]), but not when East Asian strains only were considered (data not shown).

It was previously demonstrated that *homB *and *homA *can be present in a single- or two-copy form within a genome [7]. In the present study, the *homB*/*homA *copy number was determined for all 372 strains carrying *homB *or *homA *only. All of the East Asian strains carried the single-copy genotype, and this genotype was also the most frequently found in strains isolated in Portugal (60/100, 60%), France (23/33, 69.7%), Sweden (18/22, 81.8%), Germany (15/19, 78.9%) and Colombia (11/18, 61.1%). Due to the high prevalence of the single-copy genotype, no correlation was found between *homB*/*homA *copy number and clinical outcome in these populations. Regarding strains isolated in the USA, 52.2% (12/23) were found to carry the single-copy genotype, while the remaining carried the two-copy genotype, however the distribution of these genotypes was similar among PUD and NUD strains (data not shown). Finally, concerning strains from Brazil, the two-copy genotype was the most frequently detected (28/37, 75.8%), in both PUD and in NUD strains.

When considering the group of strains isolated from Western young adults (age < 40 years, 31 PUD and 28 NUD), a correlation was observed between copy-number of a specific gene and the clinical outcome. Thus, the *homB *two-copy genotype was the most frequently observed among PUD strains and the rarest genotype among NUD strains (38.7% vs 14.3%, p = 0.069), while the inverse situation was observed for the *homA *single-copy genotype (NUD vs PUD: 35.7% vs 9.7%, p = 0.036, OR = 5.19, 95%CI [1.09–27.87]).

### Distribution of *cagA*, *vacA *s and *babA *according to clinical outcome

Considering all strains and similarly to *homB*, both *cagA *and *vacA *s1 were independently correlated with PUD (78.9 vs 65.0%, p = 0.014, OR = 2.0, 95%CI [1.16–3.47] for *cagA*; 76.8 vs 67.1%, p = 0.070, OR = 1.6, 95%CI [0.97–2.70] for *vacA*), a tendency also observed in Western strains (72.2 vs 54.6%, p = 0.014, OR = 2.2, 95%CI [1.20–3.86], and 71.0 vs 57.4%, p = 0.039, OR = 1.8, 95%CI [1.05–3.12], respectively). With regard to *babA*, it was only slightly more prevalent in PUD than in gastritis, considering all strains (58.1 vs 54.3%), and the Western strains (47.6 vs 40.7%). East Asian strains were all *vacA *s1, *cagA *and *babA*-positive. Considering the analysis by country, only the *cagA*-positive genotype was significantly associated with PUD in strains from Portugal (Table 2).

### Association of *homB *and *homA *with *cagA*, *vacA *s and *babA*

The association of *homB *and *hom*A with the *H. pylori*-virulence genotypes *cagA*, *vacA *s1 and *babA *was also evaluated. Considering only the Western strains, the presence of *homB *was associated with *cagA *(p = 0.043) and *vacA *s1 (p < 0.001), while *homA *was more frequently found in strains lacking *cagA *(p = 0.062) and with the *vacA *s2 genotype (p < 0.001). The East Asian strains were all *cagA*-positive, *babA*-positive and *vacA *s1, among which 94.2% of the Japanese isolates and 78.5% of Korean strains were also *homB*-positive.

## Discussion

Using a large panel of *H. pylori *strains (n = 372) isolated from patients from East Asian and Western countries, it was possible to confirm the association of *homB *with PUD and *homA *with NUD, previously observed with *H. pylori *strains (n = 84) isolated from Portuguese patients [7]. Considering the distribution according to geographical region, *hom*B was found to be significantly associated with PUD in strains from Western countries. However, when considering each country individually, only a tendency was observed probably due to the small number of strains tested in each case. The most common *H. pylori *strain circulating in the East Asia was extremely virulent, harboring *homB*, *cagA*, and *babA *genes and the *vacA *s1 genotype, regardless of the clinical outcome. Consequently no association with a specific disease was found, confirming the results from previous studies [8,9]. Indeed, exposure to risk factors must be heterogeneous to find an association, and this is not the case in Asia. In addition, other environmental factors, e.g. diet, and possibly host genetic factors, may contribute to this evolution [10].

The previously reported significant association of *homB *with PUD in young adults (age < 40 years, 32 patients) [7] was confirmed in the present study with a higher number of patients (n = 90).

Polymorphism in copy number of *H. pylori *OMPs may contribute to increase the fitness of the strain and also its virulence [4,11,12]. Indeed, the *homB *two-copy genotype was associated with an increased rate of *in vitro *interleukin-8 secretion as well as an increased *in vitro *adherence [7]. Furthermore, it was the genotype most frequently found in strains from young adults with PUD, while *homA *single-copy was the most frequent in NUD strains, in agreement with previous data [7]. Globally, these data suggest that in some populations, the severity of *H. pylori*-associated disease in younger subjects may be closely related to the virulence of the strain, irrespective of the contribution of host and/or environmental factors which play a major role in adults. On the other hand, the present study demonstrates that there is a marked geographical specificity regarding *homB*/*homA *copy number, particularly evident between East Asian and Western strains, but also amongst Western countries, suggesting that copy number of the *homB*/*homA *OMP coding genes also plays a role in adaptation to the human host.

Several *H. pylori *genes encoding OMP display allelic variation, as is the case of *babA*, *babB *[13], *hopQ *[14] and *hopZ *[15]. In all of these cases, a conserved profile of gene segmentation is observed, with a variable region which defines the existence of at least two highly conserved allelic variants. Regarding *homB *and *homA*, no information on allelic variation is available to date. Further sequence analysis of these coding regions using *H. pylori *strains with different geographical background would allow assessing the existence of allelic variation and to evaluate whether different alleles could be associated with a specific clinical outcome and/or reflect a dissimilar geographical origin.

The *cagA*-positive and *vacA *s1 genotypes were independently associated with PUD in Western strains, but not *babA*. Previous publications reported a significant association between the presence of *babA *and PUD in Western strains [16,17], contrasting with the present result. This discrepancy may be explained by the very heterogeneous Western study group with regard to the geographical origin of the strains, and also because of a possible absence of PCR amplification due to diversity within *babA *[17,18].

*homB *was found to co-exist with the most virulent genotypes, while *homA *was more frequently found in strains lacking these genotypes, in agreement with previous results [7]. Thus, it is likely that the phenotype resulting from the expression of *cagA*, *vacA *s1 and *homB *genes confers a biological advantage to the strain, with the cumulative action of each factor contributing at the same time to the fitness of the strains *in vivo *and to a more pronounced pro-inflammatory response. Another hypothesis would be that *homB *is linked to PUD only because of its association with other virulence factors. However, its role in *H. pylori*-associated inflammation and in bacterial adherence supports the hypothesis that *homB *contributes to disease development [7].

Globally, these results suggest that *homB *and *homA *seem to be good candidates for the pool of *H. pylori *factors involved in the determination of clinical outcome.

## Methods

### Bacterial strains

A total of 415 *H. pylori *strains isolated from patients from 10 different countries, suffering from NUD (n = 182), PUD (n = 233), of which 197 duodenal ulcers and 36 gastric ulcers, were included in this study (Table 1). *H. pylori *strains were cultured from gastric biopsies on agar supplemented with 10% horse blood, preserved in trypticase soy broth supplemented with 20% glycerol and maintained at -80°C until used. Genomic DNA was extracted from a 48 h-old culture grown in agar base supplemented with 10% horse blood, using the QIAamp DNA mini kit (Qiagen GmbH, Hilden, Germany), according to the manufacturer's instructions.

### Genotyping of *homB*, *homA*, *cagA*, *vacA *s and *babA *by PCR and sequencing

The *homB *and *homA *genes were amplified by a single PCR with a set of primers designed on a consensus internal sequence present in both genes [19]. In order to determine the *homB *and *homA *copy number, primers targeting the respective loci where used, as previously described [19]. The presence of the *vacA *s allelic variants, s1 and s2, and *cagA *and *babA *genes were determined using published PCR primers [13,16,20,21].

### Statistical analysis

Statistical analysis was performed using the statistical software package SPSS (version 14.0; SPSS). The level of significance was set at 5%, with the null hypothesis rejected when p < 0.05.

## Abbreviations

(PUD): Peptic ulcer disease; (GU): gastric ulcer; (DU): duodenal ulcer; (NUD): non-ulcer dyspepsia; (OMP): outer membrane protein; (OR): odds ratio; (CI): confidence interval.

## Competing interests

The authors declare that they have no competing interests.

## Authors' contributions

MO carried out the experimental design of the study, statistical analysis and co-drafted the manuscript; RC, YY and DQ carried out bacterial cultures and PCR; FM co-drafted the manuscript; LM supervised the study and AM supervised the study and co-drafted the manuscript. All authors read and approved the final manuscript.

## References

[B1] NIH Consensus Development Panel (1994). *Helicobacter pylori *in peptic ulcer disease. JAMA, ed NIH Consensus Development Panel on Helicobacter pylori in peptic ulcer disease.

[B2] Parsonnet J, Friedman GD, Vandersteen DP, Chang Y, Vogelman JH, Orentreich N, Sibley RK (1991). *Helicobacter pylori *infection and the risk of gastric carcinoma. N Eng J Med.

[B3] Stolte M, Bayerdörffer E, Morgner A, Alpen B, Wündisch T, Thiede C, Neubauer A (2002). *Helicobacter *and gastric MALT lymphoma. Gut.

[B4] Ilver D, Arnqvist A, Ögren J, Frick I-M, Kersulyte D, Incecik ET, Berg DE, Covacci A, Engstrand L, Boren T (1998). *Helicobacter pylori *adhesin binding fucosylated histo-blood group antigens revealed by retagging. Science.

[B5] Mahdavi J, Sonden B, Hurtig M, Olfat FO, Forsberg L, Roche N, Angstrom J, Larsson T, Teneberg S, Karlsson KA, Altraia S, Wadström T, Kersulyte D, Berg DE, Dubois A, Petersson C, Magnusson KE, Norberg T, Lindh F, Lundskog BB, Arnqvist A, Hammarstrom L, Boren T (2002). *Helicobacter pylori *SabA adhesin in persistent infection and chronic inflammation. Science.

[B6] Yamaoka Y, Kikuchi S, El-Zimaity HM, Gutierrez O, Osato MS, Graham DY (2002). Importance of *Helicobacter pylori oipA *in clinical presentation, gastric inflammation, and mucosal interleukin 8 production. Gastroenterology.

[B7] Oleastro M, Cordeiro R, Ferrand J, Nunes B, Lehours P, Carvalho-Oliveira I, Mendes AI, Penque D, Monteiro L, Megraud F, Menard A (2008). Evaluation of the clinical significance of *hom *B, a novel candidate marker of *Helicobacter pylori *strains associated with peptic ulcer disease. J Infect Dis.

[B8] Maeda S, Ogura K, Yoshida H, Kanai F, Ikenoue T, Kato N, Shiratori Y, Omata M (1998). Major virulence factors, VacA and CagA, are commonly positive in *Helicobacter pylori *isolates in Japan. Gut.

[B9] Mizushima T, Sugiyama T, Komatsu Y, Ishizuka J, Kato M, Asaka M (2001). Clinical relevance of the *babA2 *genotype of *Helicobacter pylori *in Japanese clinical isolates. J Clin Microbiol.

[B10] Tsugane S (2005). Salt, salted food intake, and risk of gastric cancer: epidemiologic evidence. Cancer Sci.

[B11] Alm RA, Bina J, Andrews BM, Doig P, Hancock RE, Trust TJ (2000). Comparative genomics of *Helicobacter pylori*: Analysis of the outer membrane protein families. Infect Immun.

[B12] Solnick JV, Hansen LM, Salama NR, Boonjakuakul JK, Syvanen M (2004). Modification of *Helicobacter pylori *outer membrane protein expression during experimental infection of rhesus macaques. Proc Natl Acad Sci USA.

[B13] Pride DT, Meinersmann RJ, Blaser MJ (2001). Allelic variation within *Helicobacter pylori babA *and *babB*. Infect Immun.

[B14] Cao P, Cover TL (2002). Two different families of *hopQ *alleles in *Helicobacter pylori*. J Clin Microbiol.

[B15] Peck B, Ortkamp M, Diehl KD, Hundt E, Knapp B (1999). Conservation, localization and expression of HopZ, a protein involved in adhesion of *Helicobacter pylori*. Nucl Acids Res.

[B16] Gerhard M, Lehn N, Neumayer N, Boren T, Rad R, Schepp W, Miehlke S, Classen M, Prinz C (1999). Clinical relevance of the *Helicobacter pylori *gene for blood-group antigen-binding adhesin. Proc Natl Acad Sci USA.

[B17] Olfat FO, Zheng Q, Oleastro M, Voland P, Boren T, Karttunen R, Engstrand L, Rad R, Prinz C, Gerhard M (2005). Correlation of the *Helicobacter pylori *adherence factor BabA with duodenal ulcer disease in four European countries. FEMS Immunol Med Microbiol.

[B18] Oliveira AG, Santos A, Guerra JB, Rocha GG, Rocha AMC, Oliveira CA, Cabral M, Nogueira A, Queiroz DMM (2003). *babA *2- and *cag*A-positive *Helicobacter pylori *strains are associated with duodenal ulcer and gastric carcinoma in Brazil. J Clin Microbiol.

[B19] Oleastro M, Monteiro L, Lehours P, Megraud F, Menard A (2006). Identification of markers for *Helicobacter pylori *strains isolated from children with peptic ulcer disease by suppressive subtractive hybridization. Infect Immun.

[B20] Atherton JC, Cao P, Peek RM, Tummuru MKR, Blaser MJ, Cover TL (1995). Mosaicism in vacuolating cytotoxin alleles of *Helicobacter pylori *– Association of specific *vac*A types with cytotoxin production and peptic ulceration. J Biol Chem.

[B21] Tummuru MKR, Cover TL, Blaser MJ (1993). Cloning and expression of a high-molecular-mass major antigen of *Helicobacter pylori*: evidence of linkage to cytotoxin production. Infect Immun.

